# Innovative financial mechanisms and investments to drive change in the global beef sector

**DOI:** 10.1093/af/vfag032

**Published:** 2026-06-25

**Authors:** Charlotte Talbott, Sara Valencia-Salazar, Gonzalo Munoz Anton, Diego Gauna, Josefina Eisele

**Affiliations:** RaboResearch Rabobank Chesterfield, Missouri 63017, United States; International Livestock Research Institute Nairobi 00100, Kenya; Productivity, Trade, and Innovation Sector/Agriculture and Rural Development Division Inter-American Development Bank Washington, D.C. 20577, United States; Inter-American Institute for Cooperation on Agriculture Lago Sul, Brasília 71600-500, Brazil; Global Roundtable for Sustainable Beef

**Keywords:** agrifood systems finance, beef system resiliency, blended finance, risk mitigation, rural economy, sustainable finance

Implications

- Rather than cataloguing financial instruments, this perspective examines why financing mechanisms have struggled to scale in beef systems and shows that these limitations are primarily driven by weak enabling conditions; as a result, sustainable finance can only deliver meaningful impact when supported by such conditions, rather than through financial instruments alone.
- Small and medium producers represent the greatest opportunity for sector‑wide sustainability gains, but they remain the least served by existing financial mechanisms. Targeted risk‑sharing tools, concessional capital, and technical support are essential to close this gap.
- Coordinated public–private approaches, blended finance, and traditional bank engagement are necessary to mobilize capital at a scale that will drive sustainability in the global beef production system.

## Introduction

Access to capital is a critical lever for transformation in the beef sector toward sustainability, resiliency, and efficiency. Historically, capital investments allocated to the beef sector have focused on food production and accessibility, but there is growing recognition of a broader, complex agrifood system.

Data availability regarding how beef and cattle supply chain participants finance production is limited. Traditional debt serves as a significant financial resource for farmers and ranchers in the United States (Lacy et al., 2026). In 2024, debt was held by 66% of midsize, 78% of large, and 78% of very large family farms, compared to less than 23% of small family farms. Both commercial banks (43.4%) and the Farm Credit System (37.8%) account for 80% of non-real estate loans, with the remaining 20% allocated between government agencies and non-traditional sources, such as life insurance companies, input suppliers, implement dealers, contractors, and cooperatives (Subedi and Giri, 2024).

Within the agrifood system, capital allocation can have significant long-term impacts on the environment, rural economies, and social well-being for both producers and consumers. Despite the growing availability of sustainability-oriented financial instruments, capital has not flowed at scale into beef production systems where environmental gains could be most significant.

This paper focuses on sustainable finance, defined as financial flows that integrate environmental, social, and governance (ESG) considerations into investment decisions. While broader food systems finance encompasses multiple funding flows across the value chain, this analysis focuses specifically on sustainability-oriented financial instruments and mechanisms within the beef sector. Sustainable finance instruments can be debt based (use of proceed bonds and sustainability linked bonds), equity-based, or have hybrid features (blended finance). Additionally, traditional debt flows may encompass sustainability-related projects on existing lines of credits and are not necessarily captured or reported as sustainable finance flows. For example, a beef producer may purchase and install equipment or technology under a traditional operating line of credit which improves environmental performance, but is not classified as sustainable finance directly.

The United Nations Framework Convention on Climate Change (UNFCCC, 2026) defines climate finance as local, national, or transnational financing—drawn from public, private, and alternative sources of financing—that seeks to support mitigation and adaptation actions that will address climate change. While this report focuses on sustainable finance (as defined above), the authors look to previous research regarding climate finance due to the lack of data on capital deployment related to sustainable finance specifically.

The shift from agricultural production financing to climate finance is being propelled by global agreements such as the Paris Agreement in 2015 and the 2019 United Nations Food Systems Summit. However, agrifood systems receive only 4.3% of direct climate finance globally, and livestock captures an even lower share (World Bank Group, 2024). It is estimated that less than USD 240 million in climate financing is allocated to livestock systems per year, exposing an USD 181 billion funding gap for animal proteins (Kirchherr et al., 2025). From 2018 to 2023, 79% of global climate finance was mobilized in three regions: Asia, Western Europe, and North America, highlighting a widening disparity between high- and low-flow regions (Kirchherr et al., 2025).

This article argues that the primary constraint is not the absence of financial tools, but the lack of enabling conditions critical for scaling sustainable finance and effectiveness across diverse beef production systems.

## Enabling Conditions for Scaling Sustainable Finance

The beef sector faces structural and financial barriers that limit the effectiveness of sustainable finance. Production systems vary widely across grazing, feedlot, and mixed operations, with a large share of cattle raised by small- and medium-sized producers. While conventional agricultural credit is widely available in many regions, sustainable finance instruments face a more specific constraint: a limited pipeline of projects that meet their eligibility criteria, including standardized methodologies, verifiable environmental outcomes, and consistent ESG reporting. This reflects not a lack of capital per se, but a misalignment between existing financial products and the requirements of sustainability-oriented financing mechanisms (OECD, 2025; Climate Bonds Initiative, 2026).

Financial constraints and opportunities also differ significantly across the beef value chain. Producers often face limited access to capital due to high perceived risk, low collateral, and fragmented operations, which constrain their ability to engage with performance-linked financial instruments. In contrast, processors and retailers are typically better positioned to access structured finance instruments such as sustainability-linked loans or bonds, given their scale, financial capacity, and ability to aggregate and report sustainability performance across supply chains. Sizing of sustainability-linked financial products may disproportionately benefit different segments of the beef supply chain. For example, lenders may look to aggregate beef producers to fill a large bond, which presents additional challenges. These differences shape both the applicability and scalability of sustainable finance mechanisms across the sector.

A central barrier to scaling sustainable finance lies in the difficulty of translating complex ecosystem value into investable financial structures. Rangeland-based livestock systems generate multiple value streams (including livestock production, carbon sequestration, biodiversity conservation, and water regulation), but these are rarely fully measured or captured within financial frameworks. As a result, many sustainable livestock initiatives remain below the threshold of risk despite generating significant societal value. Evidence suggests that rangeland restoration can generate substantial returns, with cost-benefit ratios ranging from USD 4 to USD 35 per USD invested, depending on context (Davies et al., 2025).

Limited capacity to measure environmental performance, particularly for enteric methane emissions and soil carbon, continues to constrain the development of performance-linked finance by increasing uncertainty and transaction costs. The absence of robust and cost-effective monitoring, reporting, and verification (MRV) systems limits the ability to quantify and verify environmental outcomes, while methodological fragmentation and evolving policy frameworks further increase uncertainty for both producers and financiers (World Bank Group, 2024; OECD, 2025).

Strengthening enabling conditions is therefore critical to improving the investment potential of sustainable livestock systems. Key priorities include the development of cost-effective MRV systems, producer aggregation models that reduce transaction costs, expanded access to technical assistance, and stable and predictable policy environments. Improved supply chain governance and corporate sustainability commitments can further enhance market signals and reduce perceived risk.

For financial institutions, greater integration of environmental performance into lending decisions is more likely where clear incentives exist, including improved risk-adjusted returns, regulatory alignment, or demand from downstream actors. In this context, blended finance structures, concessional capital, and risk-sharing mechanisms can play a critical role in improving the risk–return profile of sustainable investments and expanding access to finance for small- and medium-sized producers (World Bank Group, 2024).

## Financial Instruments Driving Change

While various financial instruments have emerged in recent years, their degrees of maturity and scalability differ substantially. The following cases illustrate how sustainable finance is being enabled and scaled in the global beef sector:

- **Sustainability-linked and transition instruments** tie borrowing costs to verified sustainability outcomes within the beef supply chain, such as reductions in greenhouse gas (GHG) emissions intensity or improved traceability. Applicability remains limited to larger actors with robust reporting and upstream supply chain visibility.
  **Case 1: Corporate participation**. Major global beef processors have been active in the sustainability-linked bond (SLB) and transition loan space. Some companies have reported the issuance of multi-year fixed-rate debt instruments, including transition bonds and SLBs. The proceeds support efforts to enhance traceability and reduce environmental risks within their supply chains. Deal structures typically require recipients to commit to measurable environmental targets, such as significant reductions in GHG emissions intensity or deforestation risk mitigation. Progress is verified through regular audits and if targets are not met, the interest rates on bonds or loans increase, thereby providing a financial incentive for performance.
  *&Green Project Examples*: Hacienda San José (HSJ) represents an investment in Colombia’s beef sector, where a USD ∼7.7 million long-term loan from the &Green Fund supports the scaling of a sustainable intensification model across up to 180,000 ha, combining increased productivity with forest conservation and restoration commitments (up to 27,000 ha). The financing is designed to link improved pasture management and herd practices with the protection and restoration of forest, demonstrating that productivity gains and conservation outcomes can be mutually reinforcing (&Green, 2026a). In Brazil, &Green’s support for Marfrig Global Foods focuses on supply-chain transition finance. Rather than financing a single asset, the investment backs corporate-level commitments to eliminate deforestation from beef sourcing by funding traceability systems, including for indirect suppliers, and promoting sustainable practices and pasture restoration across Marfrig’s supplier base. By leveraging a major processor’s scale and purchasing power, the financing helps drive sector-wide change while supporting smallholder inclusion and large-scale forest protection (&Green, 2026b).
  **Case 2: Uruguay**. Since 2022, Uruguay has issued two SLBs, raising USD 2.2 billion in the international capital market. The novelty of Uruguay’s case is that the bond incorporates a two-way interest rate structure. The bond coupon can potentially increase if the country fails to meet the targets set out in the issuance framework, while the country could benefit from a reduction in the bond coupon if it exceeds its environmental targets. As livestock emissions are almost 70% of total Uruguay GHG emissions, the bond success is linked to the reduction of emissions in the beef sector (National Climate Change Response System, 2022).
- **Multilateral and public development banks** deploy resources to mobilize sustainability-aligned agrifood investments and philanthropic resources, particularly in low- and middle-income countries (LMICs). Recent multilateral commitments indicate ambition to scale such finance (e.g. USD 1.3 trillion commitment during COP29 for LMIC funding by 2035), although evolving U.S. policy participation has introduced uncertainty regarding the durability of funding flows. According to the Global Landscape of Climate Finance Report, 6.3% of global climate finance flows (USD 121.5 billion) were provided by multilateral development financial institutions in 2023 (Kirchherr et al., 2025). Participation of the agricultural sector is low (∼7% of total; Naran et al., 2025).
  **Case 3:** The IFC’s investment in Minerva Foods demonstrates how multilateral development finance is primarily channeled toward large processors with the capacity to absorb capital and comply with ESG requirements. The investment combined equity participation and long-term loans to support regional expansion and improvements in environmental and social performance, including supply chain traceability initiatives (IFC, 2013). Evidence from IFC investments in the beef sector shows that financing is predominantly directed toward large processors and exporters, such as Minerva and Marfrig, which have the scale, governance structures, and reporting capacity required by lenders. In contrast, primary producers remain largely excluded from direct access to structured finance, highlighting persistent challenges related to risk, aggregation, and bankability. In Colombia, IFC has supported the development of sustainable beef value chains through advisory and pilot initiatives, including collaboration with vertically integrated companies such as CIALTA. These efforts have focused on improving traceability, production standards, and governance across the value chain, rather than providing large-scale direct financing, highlighting the role of technical assistance in building a pipeline of investable projects (IFC, 2019).
- **Government support programs** leverage public capital to reduce risk. These approaches demonstrate the role of aggregation mechanisms, clear eligibility criteria, and public institutions acting as risk mitigators.
**Case 4**: The U.S. Department of Agriculture Natural Resources Conservation Service has prioritized sustainability through public-private partnerships and practice payments, reaching over USD 3 billion issued to producers in 2024 (USDA, 2026). These financial assistance contact programs include the Environmental Quality Incentives Program, Conservation Stewardship Program, Regional Conservation Partnership Program, and others. Prescribed grazing, fencing, pasture planting, and brush management are common practices beef producers deployed.
**Case 5**: **RenovAGRO.** Since 2010, Brazil has had specific financing lines to promote the transition of the agricultural sector toward higher levels of environmental sustainability. The program, currently called RenovAGRO (or ABC+), offers preferential rates to producers who adopt verified low-carbon agriculture practices. Interest rates for the RenovAGRO program are significantly lower than commercial market rates due to interest rate equalization (subsidies) provided by the Federal Government of Brazil. The government subsidy is variable and depends on the gap with the basic interest rate (SELIC). For example, with a projected SELIC rate around 14.75% (as of March 2026) and RenovAGRO rates at 7–8%, the government subsidizes approximately 45–50% of the total interest cost the market would otherwise demand for that capital. In relation to livestock farming, the financing line for the recovery of degraded pastureland stands out. RenovAGRO allocated USD 1.5 billion for the 2025/2026 campaign, most of which is channeled through Banco do Brazil and the National Bank for Economic and Social Development (Harfuch et al., 2024).
- **Blended finance** is an innovative financing approach that strategically combines concessional public or philanthropic capital with commercial investment to de-risk projects, crowding in private finance for sustainability-related projects.
  **Case 6**: Launched in 2023, Eco Invest Brazil aims to reduce credit risk for green projects and broaden access to financing by supporting financial institutions in integrating sustainability criteria. The initiative, led by Brazil’s government with technical support, uses a blended finance mechanism that combines public and private capital through competitive auctions to fund the recovery of degraded pastures without expanding the agricultural frontier. This approach mobilized USD 5.5 billion to restore 1.4 million hectares across multiple Brazilian biomes, enhancing productivity, soil health, and environmental outcomes (COP30, 2025).

## Market-Building Initiatives and Tools

Market-building initiatives include multi‑stakeholder platforms that reduce investment risk and transaction costs in sustainable beef by aligning demand signals, standards, technical assistance, and blended finance, thereby mobilizing private capital at scale toward producers transitioning to deforestation‑free, low‑emissions, and socially responsible supply chains.

### Carbon finance as a complementary tool

Carbon finance has gained interest as a revenue source for beef producers, but its impact on sector-wide transformation remains limited. Credits for beef producers make up a small portion of voluntary carbon market retirements, with enteric methane credits only recently introduced. Gold Standard launched a beef-specific methodology in July 2023 and Verra’s enteric beef feedlot module is on hold (Gold Standard, 2023; Verra, 2024). Beef manure projects are uncommon compared to dairy and hog anaerobic digestion systems (in the United States, only nine beef digesters compared to 343 dairy and 50 hog digesters; EPA, 2024). Some producer payments and insetting protocols exist, but robust MRV requirements increase costs, especially for grazing systems. Due to high transaction costs, uncertainty, and lengthy crediting timelines, carbon finance is best viewed as a supplementary incentive within broader investment and support packages, not a standalone solution.

**Example 1**: Innovative Finance for the Amazon, Cerrado and Chaco (IFACC) does not operate as a financial institution but rather as a multi-stakeholder platform that mobilizes and coordinates private and public actors to scale investment in deforestation-free supply chains. In Brazil, Argentina, and Paraguay, over USD 950 million in farm loan products, corporate debt instruments, farmland investment funds, and capital markets offerings have been disbursed for soy, cattle, agroforestry, and non-timber forest products through a coalition of companies, banks and investors (IFACC, 2026). Its role highlights that scaling sustainable finance depends not only on financial instruments, but also on market coordination, pipeline development, and alignment of incentives across actors.

**Example 2**: In 2023, Agoro Carbon Alliance announced USD 15 million in carbon payments to farmers and ranchers in the United States, estimating 5 million tons of carbon will be sequestered over 10 years in 24 states. The model offers carbon prepayments and carbon performance-based payments to beef producers for changes to grazing management, biodiversity, and pasture fertilization (Agoro Carbon Alliance, 2023).

## Conclusions

Figure 1 illustrates how scaling sustainable finance in beef systems depends on aligning risk reduction, project viability, enabling environments, and capital alignment, with economic viability as a critical condition for long-term investment and scalability. Expanding blended finance and concessional capital can support reduced risk and expanded financial access for the beef supply chain. Under consistent MRV frameworks, producer aggregation and technical assistance for small and medium operators can be strengthened. Clear policy alignment and capacity building can enhance sustainable finance readiness in emerging markets. Aligning public and private finance can ensure complimentary incentives and durable investment structures. Traditional lending pathways that support sustainability in the beef industry point to a clear need for targeted data collection and reporting to understand how existing agricultural financing supports sustainable development. Finally, there is a need for further collaboration between public and private finance, traditional lending, cattle producers, and the broader beef supply chain to drive these enablers of sustainable finance.

**Figure 1.**
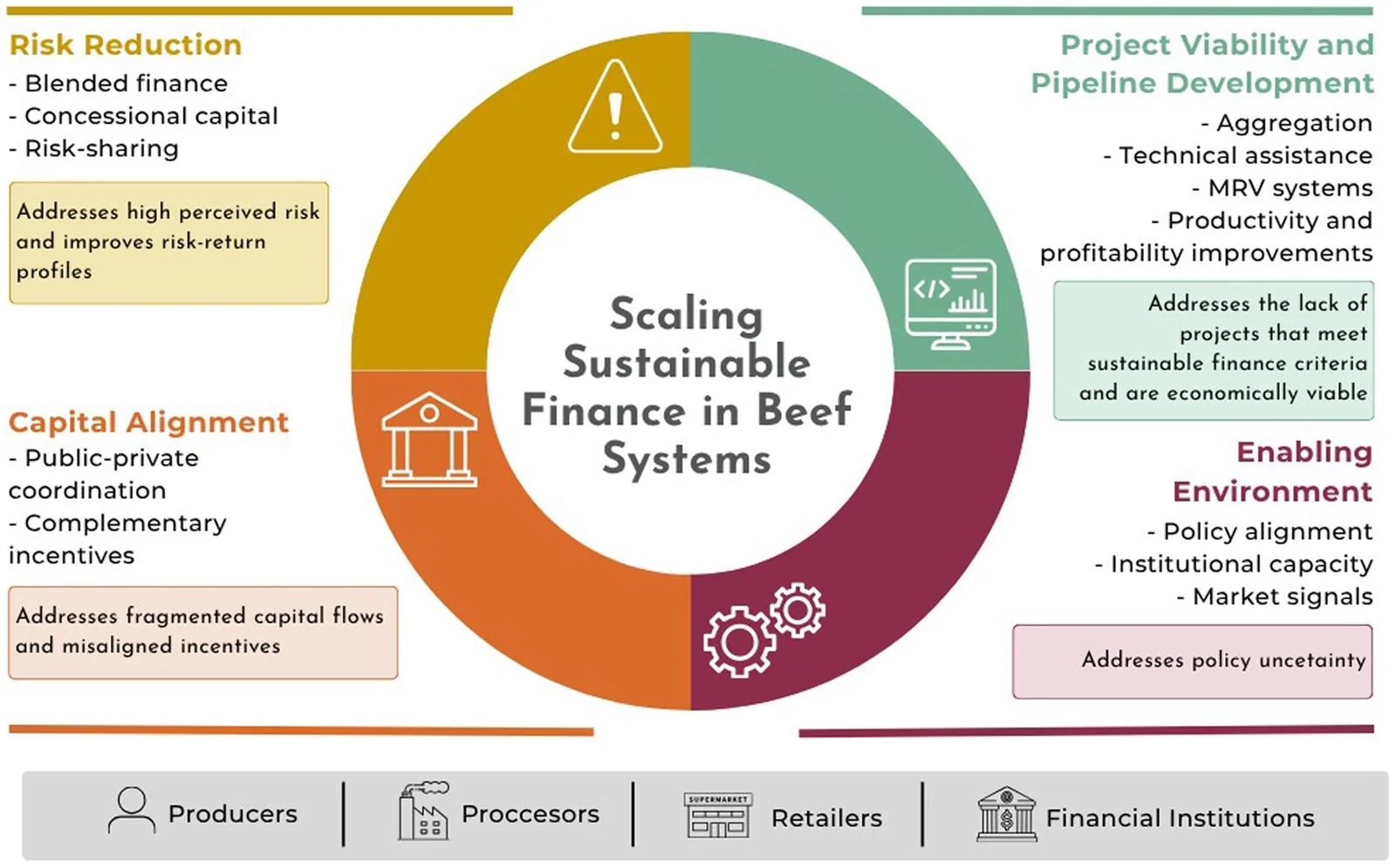
Model for scaling sustainable finance across the beef value chain.

Scaling project finance and adopting innovative technologies are essential for future progress in intensive cattle production and the beef supply chain. Financial tools like sustainability-linked debt, corporate insetting, and incentives can help meet sustainability goals, but require coordinated efforts. In Latin America, boosting efficiency, competitiveness, and resiliency in beef production depends on infrastructure investments, enabling policies, access to financing, and technical support. In the United States, investment in sustainable genetics, precision management, biosecurity, supply chain partnerships, and market initiatives are crucial for strengthening system resiliency. Aligning capital with sustainability in beef production therefore requires expanded measurement and reporting of current sustainability financing and a systemic approach and enabling conditions that account for regional and production nuances, rather than isolated financial innovation.

## Author Biographies

**Charlotte Talbott**, MS, Animal Agriculture Sustainability Analyst, Rabobank North America. She analyzes risks and opportunities associated with sustainable business development in the North American livestock industries, providing value-driven insights to clients in the animal protein and dairy sectors. Prior to joining Rabobank, Charlotte held the role of associate director of Beef Sustainability Research at the National Cattlemen’s Beef. Charlotte brings unique sector knowledge to the financial services sector from North Carolina State University, where she obtained her master’s and bachelor’s degrees in Animal Science with minors in Crop Science and Agricultural Business Management.

**Sara Valencia**, Consultant, International Livestock Research Institute. She is a sustainability specialist in tropical livestock systems with expertise in greenhouse gas mitigation and ruminant nutrition. She holds a PhD in Ecology and Sustainable Development and has contributed to the development of a Sustainable Livestock Standard and to the design of a zero-deforestation certification strategy for Colombia’s dairy and beef supply chains. She currently works on the STELARR (Sustainable Investments for Large-Scale Rangeland Restoration) project, supporting the structuring of investment frameworks to mobilize private capital toward sustainable livestock systems. She has consulted for the Colombian Ministry of Agriculture, the Alliance Bioversity–CIAT, and the International Livestock Research Institute.

**Gonzalo Munoz**, Senior Rural Development Specialist, IDB. He is a senior rural development specialist in the Agriculture and Rural Development Department of the Inter-American Development Bank (IDB). He holds a degree in agronomy and a master’s degree in finance. He has been based in Paraguay and the Dominican Republic, where he spearheaded sector policy dialogue and strategic engagement with government counterparts. Since 2017, he has been based in Washington, DC, where he has expanded his responsibilities to include oversight of the Division’s portfolio and contributions to knowledge generation and analytical work. He is currently leading the design and implementation of the Sustainable Livestock Platform for the Americas, an initiative aimed at advancing sustainability, productivity, and climate resilience in the livestock sector.

**Diego Gauna**, Assistant Professor (Agronomy Sciences, University of Buenos Aires) and Consultant, Interamerican Institute for Cooperation on Agriculture (IICA). He is an economist specializing in the intersection of agricultural economics, agricultural policy, and sustainable livestock. He has 20 years of experience in managing national and international projects related to agriculture and food. His latest works focused on driving the sustainable transformation of agrifood systems across Latin America and the Caribbean. He served on the coordination team for the Sustainable Cattle Platform for the Americas, an IICA-IDB initiative supporting a regional strategy for sustainable cattle. He holds a Licenciatura en Economía from National University of Cordoba (Córdoba, Argentina), a master’s degree in finance from San Andrés University (Buenos Aires, Argentina), and a master’s degree in agriculture economics from Iowa State University (Iowa, United States).

**Josefina Eisele**, Directo for Latin America, Global Roundtable for Sustainable Beef. She has over 15 years of experience in sustainability and agricultural systems, spanning both the private sector and civil society. She began her career evaluating investment projects in Latin America under international standards such as the IFC’s Equator Principles. Eisele later led field projects for Solidaridad’s Southern Cone and built Peterson Control Union’s sustainability consulting division, advancing sustainable supply chains. As director for Latin America at the Global Roundtable for Sustainable Beef (GRSB), she works with diverse stakeholders to promote beef sustainability. She has spoken at over 200 conferences, contributed to certification schemes, developed sustainability strategies, and since 2022, has represented GRSB at COP meetings.
